# Risk of infections in bronchiectasis during disease-modifying treatment and biologics for rheumatic diseases

**DOI:** 10.1186/1471-2334-11-304

**Published:** 2011-11-02

**Authors:** Guillaume Geri, Sabrina Dadoun, Tach Bui, Nuria Del Castillo Pinol, Simon Paternotte, Maxime Dougados, Laure Gossec

**Affiliations:** 1 Paris Descartes University, Medicine Faculty; Assistance Publique Hôpitaux de Paris, Rheumatology B Department, Cochin Hospital, Paris France; 2 Pneumology Department, Cochin Hospital, Paris France

## Abstract

**Background:**

Bronchiectasis is frequently associated (up to 30%) with chronic inflammatory rheumatic diseases and leads to lower respiratory tract infections. Data are lacking on the risk of lower respiratory tract infections in patients treated with biologic agents.

**Methods:**

Monocenter, retrospective systematic study of all patients with a chronic inflammatory rheumatic disease and concomitant bronchiectasis, seen between 2000 and 2009. Univariate and multivariate analyses were performed to evidence predictive factors of the number of infectious respiratory events.

**Results:**

47 patients were included (mean age 64.1 ± 9.1 years, 33 (70.2%) women), with a mean follow-up per patient of 4.3 ± 3.1 years. Rheumatoid arthritis was the main rheumatic disease (90.1%). The mean number of infectious events was 0.8 ± 1.0 event per patient-year. The factors predicting infections were the type of treatment (biologic vs. non biologic disease-modifying treatments), with an odds ratio of 8.7 (95% confidence interval: 1.7-43.4) and sputum colonization by any bacteria (odds ratio 7.4, 2.0-26.8). In multivariate analysis, both factors were independently predictive of infections.

**Conclusion:**

Lower respiratory tract infectious events are frequent among patients receiving biologics for chronic inflammatory rheumatic disease associated with bronchiectasis. Biologic treatment and pre-existing sputum colonization are independent risk factors of infection occurrence.

## Background

Biologic disease-modifying treatments have introduced a new era of disease control in inflammatory rheumatic diseases. However, non biologic disease-modifying antirheumatic drugs (DMARDs) and more so, biologics, including tumor necrosis factor (TNF) inhibitors, seem to increase the risk of infectious events [1-3]. Several studies in particular issued from randomised trials, indicated that the infection rate was up to two fold higher among rheumatoid arthritis (RA) patients receiving TNF inhibitors compared with those receiving methotrexate alone. However, this increase in risk is not consistently reported [4,5]. Infections during rituximab or abatacept treatment may also be increased, although this increase was not significant in a meta-analysis [6]. Infections occurring with biologics (especially TNF inhibitors) often also concern the lower respiratory tract [3]. In patients with RA, irrespective of the treatment, infections frequently concern the respiratory tract [7] and pre-existing chronic lung disease was one of the strong predictors of infections [8].

Bronchiectasis is defined by an irreversible airway dilatation with chronic bronchial inflammation [9]. Sputum and chronic cough are the main clinical features. The diagnosis is confirmed by high resolution computed tomography scans. Patients with bronchiectasis suffer from recurrent acute exacerbations, which may require hospitalization [10,11]. The exact prevalence of bronchiectasis is unknown, [12] but probably underestimated because of the confusion with chronic bronchitis and the lack of systematic investigations. The prevalence is estimated around 1 case in 1,000 adults in the United Kingdom [13]. Bronchiectasis occurs in bronchial obstruction (broncholithiasis), bronchial stenosis from infections (tuberculosis) and foreign body aspiration but may also be the main feature of pulmonary diseases as cystic fibrosis or associated with systemic diseases (primary ciliary dyskinesia, immunodeficiency states, alpha 1 antitrypsin deficiency, inflammatory bowel disease and rheumatic diseases, in particular RA) [14,15]. The prevalence of bronchiectasis in RA has been evaluated by high resolution computed tomography in small studies [16-19]: the prevalence was very high since bronchiectasis was evidenced in 18 to 30% of patients.

Taken together, these observations suggest a potential increased risk of infections of lower respiratory tract in patients with chronic rheumatic disorders treated with biologic DMARDs. The objective of this study was to evaluate the risk of lower respiratory tract infectious events among patients followed for bronchiectasis and receiving non biologic DMARDs and/or biologic treatments for rheumatic diseases, and to assess factors associated with infections, and in particular disease-modifying treatments.

## Methods

### Study design

Monocenter, investigator-initiated, systematic retrospective study.

### Patient selection

All in and outpatients from one tertiary rheumatology department (Cochin Hospital) seen between January 2000 and July 2009 were screened through a full-text search of the computerized database of patients' files (using the key words ["rheumatoid arthritis" or "systemic erythematosus lupus" or "ankylosing spondylitis"] AND "bronchiectasis"). Data were censored before 2000 due to the absence of biologics before that date. Patients were included in the present study if (a) they had a definite diagnosis of an inflammatory rheumatic disorder (American Rheumatism Association criteria for RA [20] and for systemic lupus erythematosus [21,22], and Amor's criteria [23] for spondylarthritis), (b) they had definite bronchiectasis, and (c) they were exposed to at least one non biologic DMARD and/or biologic treatment for their rheumatic disease during at least three months with a duration of follow-up in the department, subsequent to the diagnosis of bronchiectasis.

Non biologic DMARDs included methotrexate, leflunomide, azathioprine, ciclosporine, hydroxychloroquine, sulfasalazine, penicillamine, cyclophosphamide and gold salts. Biologic DMARDs included TNF inhibitors (etanercept, adalimumab and infliximab), rituximab, abatacept and tocilizumab. Drug selection was made by the medical staff, according to usual practice and based on the most recent published data concerning rheumatic diseases management.

A diagnosis of bronchiectasis according to expert opinion based on high resolution computed tomography scan abnormalities required that at least two different airways in areas of non-consolidated lung met one or more of the following criteria [12]: (a) inner diameter of airway lumen larger than the diameter of the accompanying pulmonary artery, (b) airway visible within 1 cm of pleural edge/chest wall, (c) non-tapering of airway for at least 2 cm beyond last branch point. All patients included in the study had a high resolution computed tomography scan. Others pulmonary investigations were performed according to the physician in charge. Recent infection of lower respiratory tract before the diagnosis of bronchiectasis was an exclusion criteria because of well known transient bronchiectatic changes seen on CT scans after an episode of pneumonia.

### Global data collection

Data were collected by 2 investigators (GG and SD), based on the computerised file and if necessary the paper file, using a standardised extraction form. Data collected were: age, sex, date of diagnosis, type and characteristics of the rheumatic disease (for RA: rheumatoid factor status, anti-cyclic citrullinated peptide antibodies, and erosiveness yes/no, and for spondylarthritis, HLA B27 status). For bronchiectasis, date of diagnosis, underlying related systemic disease, tobacco consumption and pre-existing bacteriologic colonisation were collected. Sputum bacteriologic colonisation was collected at the first period of treatment of the study and during each treatment period in detail (by bacterium) and analysed as: none, or at least one bacterium. Moreover, pneumologist opinion was collected in patients' files regarding the potential relationship between bronchiectasis and the rheumatic disease. All the HRCT were reviewed with a radiologist.

### Infectious event definition and rate

An infectious event was defined as change in sputum production, increased dyspnea, increased cough, fever, increased wheezing, malaise, radiographic changes consistent with a new pulmonary process, changes in chest sounds and reduced pulmonary function [24]. Only infectious events necessitating prescription of antibiotics for pulmonary purposes were recorded. Infections in bronchiectasis are usually defined. Sputum quality was checked with Geckler classification [25]: only suptum Geckler 5 were kept.

Infectious events were reported by treatment of rheumatic disease period, as events per patient-year. Each patient could thus be analysed several times, according to the different treatments received. Infectious events were analysed by treatment period distinguishing non biologic DMARDs (pooled) versus biologics (pooled). Among biologic treatments, infectious rates were reported for each biologic for descriptive purposes but the rates were not compared statistically due to the small sample size.

### Statistical analysis

Patients' characteristics were reported as number (percentage) for categorical variables and mean ± standard deviation (SD) for continuous variables. The primary outcome was the rate of infections per patient-year. This rate was calculated as infectious events per patient-year of follow-up: the number of events was divided by (number of months of follow-up/12). The date for inclusion was defined as the first follow-up posterior to 2000, or prior to the diagnosis of bronchiectasis, as applicable. The end of study date was defined as the last follow-up date in our centre, or the censoring date (data collection, i.e., July 2009).

To evidence predictive factors of infections, 2 separate analyses by univariate then multivariate logistic regressions were performed. (a) At the patient level, the dependent variable was the number of infections per patient-year above the mean for all patients (i.e., > 0.8 infectious events per patient-year of follow-up) and explanatory variables were age, sex, rheumatic disease type and duration, bronchiectasis disease duration, and number of previous DMARDs (non biologic or biologic). (b) At the treatment period level, the dependent variable was the number of infections per patient-year above the mean for all periods (i.e., > 0.8 infectious events per patient-year of follow-up) and explanatory variables were DMARD type (non biologic DMARD versus biologic), steroid intake and steroid dose, sputum colonisation during the treatment period (yes/no) and number of previous biologic treatments. In the multivariate logistic regression analyses, all variables with a p value <0.20 in univariate analyses were entered into the model. Linear regression was also performed to find predictive factors of the number of infectious events. Results were similar to the logistic regression results (data not shown). For all statistical analyses, a p-value less than 0.05 was considered statistically significant. Statistical analyses involved use of the SAS release 9.1 statistical software package.

## Results

### Selection process and follow-up

**Figure 1:**
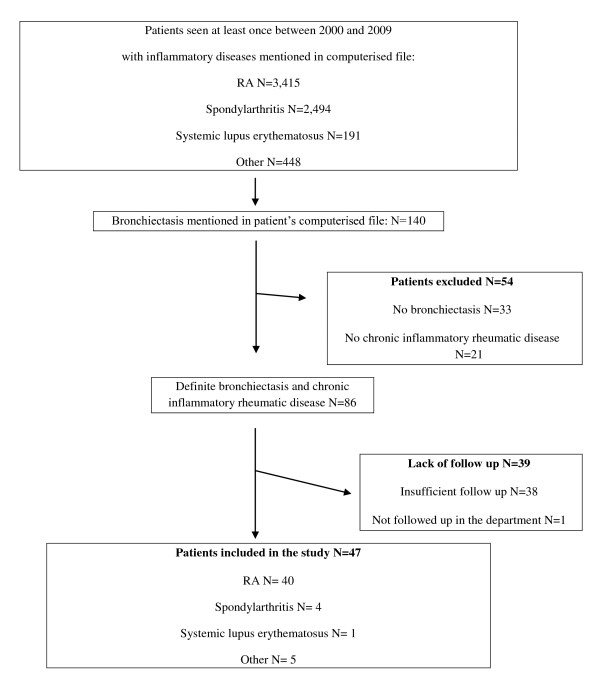
**Flow Chart of patient selection to collect all patients in one centre treated with either DMARDs or biologics for a rheumatic disease, and with concomitant bronchiectasis, with a follow-up of at least 3 months in our centre**.

Among the 6,548 patients seen in the department between 2000 and 2009 for suspected inflammatory rheumatic diseases, bronchiectasis was mentioned in the computerized file in 140 cases (Figure 1). Finally, 47 patients were included in the study (Figure 1). These 47 patients totalised 98 periods of treatment with a mean duration of treatment period of 2.1 ± 2.2 years. The mean follow-up per patient was 4.3 ± 3.1 years (range, 3 months to 13.5 years); thus, the total data concerned 194 patient-years of follow-up.

### Baseline characteristics of patients (Table 1)

**Table 1:** Baseline characteristics of 47 patients followed-up in one center for a rheumatic disease necessitating DMARD and/or biologic therapy, and with definite bronchiectasis

	All patients (N = 47)	Rheumatoid Arthritis (N = 40)	Others diagnosis (N = 7)
Mean age (SD) [range], years	64.1 (9.1) [38;78]	65.8 (7.9) [43;78]	54.4 (9.7) [38;67]
Sex N (%) female	33 (70.2)	30 (75.0)	3 (42.9)
Mean disease duration of rheumatic disease at first follow up (SD) [range], years	10.7 (8.8) [1;43]	11.5 (8.9) [1;43]	6 (7.2) [1;19]
Disease duration of bronchiectasis at first follow up, years, mean (SD), [range]	6.4 (15.9) [0;68]	7.5 (17.3) [0;68]	1.7 (3.7) [0;10]

NA: not applicable

Percentages are presented as % of available data

For the 47 patients included, mean age at inclusion was 64.1 ± 9.1 years and 33 (70.2%) were females.

The main underlying rheumatic disease was RA (40 patients, 85.1%), 4 (8.5%) had spondylarthritis; 1 (2.1%) had systemic lupus erythematosus and 5 (10.6%) had another underlying rheumatic disease (2 patients with SAPHO [synovitis, acne, palmoplantar pustulosis, hyperostosis, and osteitis] and 3 patients with an unclassified inflammatory rheumatic disease). Among the RA patients, 27 (79.4% of available data) had typical radiographic erosions. Anti-cyclic citrullinated protein antibodes and rheumatoid factor were noted in 12 (80% of available data) and 35 (100% of available data) patients, respectively. Among the 4 spondylarthritis patients, 3 were HLA B27 positive (100% of available data).

Bronchiectasis mean duration at inclusion in the study for all patients was 6.4 ± 15.9 years (range 0; 68 yrs), 27 (60%) appeared after the rheumatic disease, and 13 (37% of available data) were related to RA. Bronchiectasis not related to RA was due to infectious respiratory events in childhood in all cases. No case of cystic fibrosis or others congenital disorders were observed. Bronchiectasis was described on high resolution computed tomography as diffuse in all cases but 2 (95.7%) patients. Bronchiectasis was moderate in most patients (40/47; 85%); it was associated with other radiological patterns i.e., bronchial wall thickening and bronchiolitis signs (air trapping, centrolobular micronodules) in 10 and 6 cases, respectively. No specific pharmacologic treatment was needed for bronchiectasis, before the diagnosis of rheumatic disease. Five patients had bacteriologic colonisation during the first period of DMARD treatment: there was 1 single colonisation with *Pseudomonas aeruginosa*, 1 single colonisation with *Staphylococcus aureus *and 1 with *Haemophilus Influenzae; *2 patients had mixed colonisation (*Pseudomonas aeruginosa *and *Staphylococcus aureus *in one case and *Pseudomonas aeruginosa *and *Haemophilus Influenzae *in the other case). Colonisation appeared for the first time at the second period of treatment in one patient (*Pseudomonas aeruginosa*) and at the third period of treatment in one patient (*Pseudomonas aeruginosa *and *Haemophilus Influenzae)*. New bacteria were found in already positive sputum patients in 2 cases. No colonisation was noted in 61 periods, i.e., 32 patients. Data were not available in 19 periods (6 patients). All patients were screened for mycobacterial infections: none was evidenced in the study. Tobacco consumption was declared by 12 (25.5%) patients: 4 (33.3%) were current smokers. Smoking was given up for 22 (15; 28) years in ex-smokers patients.

Among the 98 periods of treatment, there were 58 periods of biologic treatment (Table 2) and 40 periods of non biologic DMARD treatment (16 with methotrexate alone, 16 with leflunomide alone and 8 with associations or other non biologic DMARDs including hydroxychloroquine, sulfasalazine, penicillamine, azathioprine, cyclophosphamide, gold salts, cyclosporine). The median dose of corticosteroids was similar in the patients with lower respiratory tract infections compared to those without lower respiratory tract infections (6 [5-9.5] vs. 7 [5-10] mg/d; p = 0.67).

**Table 2:** Lower respiratory tract infections in bronchiectasis concomitant to inflammatory diseases, according to the rheumatic disease-modifying treatment

	All treatments	Non-biologic DMARDs	Biologic DMARDs	Etanercept	Adalimumab	Infliximab	Abatacept	Rituximab
**N periods of treatment of the rheumatologic disease**	98	40	58	19	4	12	10	9
**Total N infections**	93	16	77	13	3	34	23	4
**N patient-years of follow-up**	194	98	96	33	1	30	17	12
**N infections per patient-year, mean (SD) [95% CI]**	0.8 (1.4) [0-3.6]	0.2 (0.5) [0-1.2]	1.2 (1.6) [0-4.3]	0.8 (1.4) [0-3.5]	2.3 (2.1) [0-6.4]	1.9 (1.6) [0-5.0]	1.9 (1.9) [0-5.6]	0.3 (0.7) [0-1.7]

One patient was treated by anakinra ans is not detailed in the table

### Infection rate and predictive factors

Among the 47 patients, a total of 93 infections was noted, i.e., a mean of 0.8 ± 1.0 infectious events per patient-year of follow-up for all patients (range 0-4.3). Nine cases of respiratory tract infections occurring in 8 patients necessitated hospitalization (of whom, one patient was hospitalized in an intensive care unit for acute respiratory distress). In all the other cases, the infection was treated in outpatients. Most of the infectious events 92/93 (98.9%) occurred in RA patients. Bacterium causing infectious events of the lower respiratory tract were the same previously found in pre-existing colonized sputum.

At the patient level, no variable (age, sex, underlying rheumatic disease type and duration, bronchiectasis disease duration and number of previous DMARDs) was predictive of infections. Analyses were performed to explain the number of infectious events per period: above or below the mean (0.8 per treatment period). In univariate analysis, only 2 factors were predictive of infections. Biologics were associated with more infections than non biologic DMARDs (1.2 ± 1.6 vs. 0.2 ± 0.5 infections per patient-year; p = 0.001). Colonisation at the beginning of the study period was also predictive of infections (0.5 ± 1.0 vs. 2.5 ± 1.7 infections per patient-year; p = 0.0001). In multivariate analysis, both factors were independently predictive of infections (Table 3). The odds ratio for infections with versus without biologics was 8.7 (95% confidence interval, CI: 1.7-43.4) and 7.4 (95% CI: 2.0-26.8) for infections with versus without colonisation. Among the biologics, infections appeared less frequent with etanercept and rituximab (Table 2).

**Table 3:** Predictive factors of respiratory tract infections in multivariate logistic regression.

	Odds Ratio (95% CI)	P value
**Bacteriologic colonisation**	7.4 (2.0-26.8)	0.002
**Treatment with biologics** **(vs non biologic DMARDs)**	8.7 (1.7-43.4)	0.008

## Discussion

In the present study, the rate of infections was higher with biologics than with non biologic DMARDs (odds ratio 8.7, 95% CI 1.7-43.4). The second factor predicting infections was sputum colonisation (odds ratio, 7.4, 95% CI 2.0-26.8). The increased risk of infectious events during biologic treatment has been previously described but the originality of this study is to analyse infections in rheumatic patients with a concomitant condition, i.e., bronchiectasis which is frequent in chronic rheumatic disorders.

First, because of the high reported prevalence of bronchiectasis in RA, and because of the infectious risk incurred with biologics in case of bronchiectasis, the present results suggest systematic clinical screening for bronchiectasis before introduction of a biologic. In case of evocative respiratory symptoms related to bronchiectasis after clinical questioning and examination, screening with a high resolution computed tomography may be indicated. Systematic high resolution computed tomography scans before introduction of biologics whatever the existence of pulmonary symptoms cannot be supported to date because our data are not strong enough to support such a position. This hypothesis should be discussed after further research on the natural history of asymptomatic bronchiectasis associated to chronic inflammatory rheumatic diseases. Indeed, if an infectious risk was confirmed to be increased in these patients, high resolution computed tomography scans should be discussed in each patient before introduction of biologics.

The second important implication of this work concerns treatment choices in patients with a rheumatic disease and concomitant bronchiectasis. These results suggest that patients with chronic inflammatory rheumatic disease and concomitant bronchiectasis should be preferentially treated with non biologic DMARDs, rather than biologics, where the rheumatologic situation makes such a choice acceptable. When biologics are necessary, considerations on infections are important to take into account. Etanercept might currently be the biologic to consider first in patients with concomitant bronchiectasis. Indeed in the present study, among biologics, etanercept and rituximab appeared to lead to less infection than other biologics. Less data are available for infections during rituximab treatment [3,6] but from a physiological point of view, this immunosuppressive drug probably also increase the risk of infections. Indeed, several studies of rituximab [26,27] indicated a slightly higher infection rate in treated patients; the most common infections were upper and lower respiratory tract infections, nasopharyngitis and bronchitis. However the sample size is small and cannot demonstrate any differences between molecules in a same therapeutic class but other larger studies have indicated seemingly lower rates of infections with etanercept [28]. In any case, whatever the treatment choice, respiratory tract infections should be carefully monitored in these patients.

In the present study, bacteriologic colonisation was an independent predictive factor of lower respiratory tract infections. This finding is consistent with previous reports showing bacteriologic colonisation as a predictive factor of lower respiratory tract infections in the following year for patients with chronic obstructive pulmonary disease (odds ratio 6.3) [29]. Therefore, these results suggest patients with bronchiectasis should be regularly screened for colonisation by sputum examinations. Indeed, knowledge of bacteriologic colonisation may lead to different antibiotic choices in case of infectious events. Furthermore, *Pseudomonas *colonisation should be carefully considered as it is a predictive factor for pulmonary function decline [30], as is the case in bronchiectasis related to cystic fibrosis [31,32].

The infectious risk in the global population of the study is lower than that described previously: infections in bronchiectasis have been reported to occur with an incidence of 1.5 to 7 events per patient-year [24,33,34] whereas in the present study, the rate was 0.8 ± 1.0 infections per patient-year. Reasons for the lower rate in the present study may be multiple. Firstly, it is possible that the retrospective nature of the study led to an underestimation of colonizations and/or of infections. It is noteworthy that the rate of colonization was low in the present study. However, all sputum tests performed for our patients were carefully analysed in a tertiary-care hospital bacteriology unit. Secondly, systematic vaccinations in our department for pneumococcal and influenza infections may improve airway disease control. These vaccinations are recommended before introduction of biologics [35] but also in patients with bronchiectasis [36]. Thus, these findings suggest to check vaccination status in those patients. Finally, systematic and very regular follow-up of our patients for their rheumatic disease can contribute to a lower rate of lower respiratory tract infections, possibly through prescription of pulmonary rehabilitation. Indeed, pulmonary rehabilitation is a well-established and effective intervention for patients with bronchiectasis leading to reductions in the incidence of acute exacerbations and reduced health care utilisation as well as improvements in exercise tolerance and health-related quality of life [37,38].

Conclusions of the present study should be interpreted with cautions because of limitations related to the study design: the retrospective nature of the study and the small sample size due to the low prevalence of the disease. Along this line, comparisons between biologics should be looked at with the greatest caution.

## Conclusions

This study indicates factors predicting respiratory tract infections in patients with chronic inflammatory rheumatic disease associated with bronchiectasis were biologic treatments and sputum colonisation. Considering on one hand the prevalence of bronchiectasis in RA, and on the other hand the increase of biologics' prescription, physicians should be careful about any respiratory symptoms before initiating biologics and use of high resolution computed tomography may be warranted in certain cases. In case of definite bronchiectasis, prescription of biologics should be carefully weighted. In such a case, careful monitoring of respiratory infections is essential and should include systematic sputum bacterial examination to search for sputum colonisation. Larger and prospective studies are needed to better assess treatment options, screening and monitoring in these patients.

## Conflict of interest statement

The authors declare that they have no competing interests.

## Authors' contributions

GG and LG designed the study. GG, SD and NDC collected the data. SP and LG performed the statistical analysis. MD, TB and LG reviewed the manuscript. All authors read and approved the final manuscript

## Pre-publication history

The pre-publication history for this paper can be accessed here:

http://www.biomedcentral.com/1471-2334/11/304/prepub
